# Impact of Organic Manure on Growth, Nutrient Content and Yield of Chilli Pepper under Various Temperature Environments

**DOI:** 10.3390/ijerph16173031

**Published:** 2019-08-21

**Authors:** Botir Khaitov, Hye Jin Yun, Yejin Lee, Farrukh Ruziev, Thi Hien Le, Mirjalol Umurzokov, Aung Bo Bo, Kwang Min Cho, Kee Woong Park

**Affiliations:** 1 Crop Science Department, Chungnam National University, Daejeon 34134, Korea; 2 Soil Management Division, NIAST, Rural Development Agency, Wanju 55365, Korea

**Keywords:** chilli pepper, organic manure, application rate, elevated temperature, rain-shelter plastic house, greenhouse, field

## Abstract

Expected climatic changes likely elicit serious challenges for crop production. Therefore, it is indispensable to investigate the response of crop growth parameters and yield under temperature variability environments. The current experiment on chilli pepper growth was conducted in a field, rain-shelter plastic house, and plastic greenhouse, with accumulated temperatures of 2832 °C, 2967 °C, and 3105 °C in 2017; and 2944 °C, 3091 °C, and 3168 °C in 2018 growing seasons. Based on soil analysis, 132.7 kg ha^−1^ (1× of livestock manure compost as an optimum and 265.4 kg ha^−1^ (2×) as a double amount of organic matter were applied to each simulated temperature condition. The results showed that organic manure application favorably affects the growth attributes and nutrient uptake of chilli pepper with the highest values found in the plastic greenhouse, followed by the rain-shelter house, over the open field cultivation condition. The highest growth of chilli pepper was at the 2× rate of organic manure application, whereas the highest yield was found at the 1× rate of organic manure application. The application of organic manure at the 1× rate in the greenhouse increased root, shoot, and fruit dry weights of chilli pepper by 21.4%, 52.4%, and 79.7%, respectively, compared to the control values. These results indicate that the rational use of organic amendments might be the best solution for chilli pepper production under variable climate conditions.

## 1. Introduction

Global warming poses a great threat to crop production all around the world. The increase in atmospheric temperature in response to global climate change might have serious consequences for crop production and be dangerous for food security [1]. Furthermore, continuously increasing populations in the world put pressure on agriculture to produce more crops. The consequences of climate change on crop production, cropping system, and soil fertility are expected to be huge but uncertain [2]. According to scientific predictions, climate change will be responsible for increase in temperature and precipitation up to 1.0–5.0 °C and 9–11%, respectively, depending on regions by the end of this century [3]. This will very likely bring a negative impact to crop production [4,5]. While, simultaneous rise in atmospheric CO_2_ and temperature are already showing some impact on nature, in the meantime, it is prudent to study the performance of crop species under simulated high temperature conditions.

The effectiveness of organic amendments is also theorized to change along with climate change [6]. Application of organic manure increases organic elements’ availability in soil, thereby improving the nutrient use efficiency (NUE) of crops and alleviating the harmful impact of climate change on crop production [7]. However, the application rate of chemical fertilizers has soared extensively during the last decades to enhance crop yield in order to meet the increasing demand of populations. The consequences are already visible on serious ecological disturbances, especially in pollution of soil and water resources. In recent years, the scientific community has been informing the advantages of implementing organic farming in agricultural production to ensure sustainable nutrient management for crops, food safety, and soil health [8]. This strategy improves NUE, while maintaining high productivity of crops, soil quality, and agricultural sustainability.

Soil is one of the most important environmental factors with indispensable significance in plant physiology [9]. Thus, maintaining soil quality is of great importance for crop growth and enhancing productivity. Organic manure is a natural source of nitrogen (N) formation process in the soil. Livestock manure returns essential macroelements including N (2.42%), P (1.51%), and K (0.41%), as well as micronutrients such as magnesium, calcium, sulfur, and manganese to the soil while maintaining its fertility. Several researchers have reported that nutrients available for plant uptake increase rapidly in the experimental soil after organic manure application, although bacterial populations were different in various soils [10,11]. Microorganisms living in the soil are important for decomposing, mineralizing, and recycling organic matters [12]. Microbial populations intensively induce the production of phytohormones such as gibberellin and auxin in plant roots grown in fertile soil with rich organic manures which stimulate plant growth [13]. 

Chilli pepper (*Capsicum annuum*), the most known species of the plant genus Capsicum (peppers), originated in Mexico, the southern part of North America. Nowadays, this crop is commonly cultivated in all continents of the world and mainly used for food and pharmaceutical uses. Superior levels of bioactive and antioxidant compounds such as carotenoids, phenolic compounds, and ascorbic acid are contained in fresh chilli pepper fruits [14]. However, the level of these components in the fruit depends on genetic and environmental factors. If chilli pepper is grown for a food ingredient, its quality and market value are determined by the level of fruit pungency [15]. Some essential components are enhanced in organically cultivated crops, for instance, quercetin (flavonoid) in spinach, Chinese cabbage, and Welsh onion [16]; phenol and flavonoid in *Labisia pumila* [17]; protein and carotene in *Capsicum chinense* [18]; and capsaicin content in pepper [11].

In Korea, the cultivation area of chili peppers is ~30 thousand hectares, and the fruit production reached ~72 thousand tones in 2018, increasing by 16 thousand tones (28.4%) from 56 thousand tones in 2017 [19]. It is well-known that climate change causes modifications in crop physiology, NUE efficacy, yield, and other parameters [20]. Therefore, it is important to elucidate the impact of climate change on crop productivity for sustainable agricultural production.

Our goal in this study was to evaluate the effect of 1× rate (132.7 kg ha^−1^) and 2× rate (265.4 kg ha^−1^) livestock manure treatments compared to control (without manure) on the growth and yield parameters of chilli peppers grown under three different environmental conditions such as a plastic greenhouse, rain-shelter plastic house, and open field conditions. The objective of this research was to study how chilli peppers react to the different rates of organic manure application under expected climate change scenarios. Furthermore, we were curious to understand how plant physiology parameters such as chlorophyll content, plant height, shoot, and root dry weights change in response to the applied organic manure under various temperature environments.

## 2. Material and Methods

### 2.1. Experimental Conditions and Plant Material

The climate of South Korea with four seasons allows the cultivation of many crop species. The average annual air temperature ranges between −5 °C to −2.5 °C in January and 22.5 °C to 25 °C in July. The annual precipitation is ~1380–1400 mm, the main part of it falls between November and May, with an average humidity of 66%.

A two-year experiment, during 2017 and 2018 growing seasons, was conducted in the field, rain-shelter plastic house, and plastic greenhouse conditions simulating three different temperature environments. Climate parameters in the plastic greenhouse and rain-shelter plastic house were recorded with an Em50^®^ series data logger (METER Group, Inc., Pullman, WA, USA). During the growing seasons, the highest air temperature was observed in the greenhouse, followed by rain-shelter plastic house compared to the field. However, the highest amount of rainfall precipitated in the field experiment. The accumulated temperatures for 130 days were 2832 °C, 2967 °C, and 3105 °C in 2017; and 2944 °C, 3091 °C, and 3168 °C in 2018 in plastic greenhouse, rain-shelter plastic house, and open field conditions, respectively. The detail climatic records for both growing season are presented in Table 1.

Seeds of chilli pepper (*Capsicum annuum* L.) variety—Keunsarang (Nongwoo Bio, Sunwon, Korea)—were obtained from Crop Physiology department, Chungnam National University, Korea. Prior to starting the experiment, the seeds were sorted: broken seeds were discarded and good quality seeds were chosen for further use in the experiment. The seeds’ surface was sterilized with a 0.2% Hg_2_Cl solution for 3 min, rinsed in distilled water thoroughly, and left to dry at room temperature (25 °C). Then, the seeds were sown directly into 2 cm depth soil in pots (30 cm in diameter). Each pot was filled with 4 kg of soil (55% clay, 20% silt, and 25% sand) and the experiment was commenced in the greenhouse of Crop Physiology Department, Chungnam National University. The seeds germinate under favorable environmental conditions in the 2nd week of April each year (day length 15–16 h, temperature 25–30 °C, and humidity 65–70%). When the germination was complete after 7 days, the percentage of seed germination was calculated and the seedling density was thinned to four seedlings per pot.

The seedlings of chilli pepper were transplanted into a plastic greenhouse, rain-shelter plastic house, and open field at the experimental station located in Unbong-eup, Namwon-si, Jeollabuk-do, Republic of Korea. The experiment was set in randomized complete block design with three replicated plots (15 m × 4 m) for each treatment.

The chemical analyses of organic manure were conducted to estimate N, P_2_O_5_, and K_2_O contents before application. The organic manure consisted of 67.06% organic matter, 2.42% N, 1.51% P_2_O_5_, and 0.41% K_2_O. Two different levels of organic matter were applied to each temperature treatments as per experimental layout. Based on a soil analysis, 132.7 kg ha^−1^ (1×) of livestock manure compost as an optimum and 265.4 kg ha^−1^ (2×) was applied as the double rate of organic manure.

Ammonium nitrate (34%), fused phosphate (17%), and muriate of potash (52%) were applied as inorganic sources of N, P, and K. Recommended doses of the chemical fertilizers N, P (P_2_O_5_), and K (K_2_O) were 80 : 50 : 40 kg ha^−1^, and this amount was divided into three equal portions. The first part was applied as a basal dose and the two remaining portions were given during the vegetation period. All other agronomic operations such as plant protection, weeding, and irrigation measures were conducted similarly in all plots.

### 2.2. Soil Analyses

The soil in the experimental area belongs to Podzolic (ash-gray forest soil), slightly acidic with pH 5.9–6.6 and EC 1.3–2.4 dS/m. It consists of clay 540–585 g kg^−1^, silt 260–291 g kg^−1^, organic matter 18.7–25.7 g kg^−1^, and sand 150–172 g kg^−1^. Soil chemical analyses indicated that NO_3_–N, 52.9 mg/kg; NH_4_^+^–N, 13.2 mg/kg; P_2_O_5_, 671.4 mg/kg; K, 1.2 cmol/kg; Ca, 8.5 cmol/kg; Mg, 2.6 cmol/kg; and Na, 0.3 cmol/kg.

Soil samples were randomly collected at 0–30 cm depth in sealable plastic bags from each replicated plot every month during the experiment. Air-dried soil samples at room temperature were ground and sieved through a 2-mm mesh before chemical analysis.

Soil and plant samples were analyzed according to the methods developed by the National Institute of Agricultural Science and Technology (NIAST, 2000). Soil pH and EC parameters were measured with pH and EC meters using 1 : 5 ratio of soil and distilled water. The organic matter was extracted using the Tyurin method, available phosphate was extracted using the Lancaster method [21], and substitutional cation was extracted using 1M NH_4_OAC (pH 7.0) and analyzed with a coupled plasma spectrophotometer (Integra XL, GBC, Toronto, Canada).

Plant materials were dried and pulverized, and then 1 mL of concentrated sulfuric acid and 10 mL of 50% perchloric acid were added into 0.5 g of the sample, followed by decomposition by heating on a hot plate. Total N, P_2_O_5_, and K_2_O were analyzed with Kjeldahl distillation, Vanadate method, and inductively coupled plasma spectrophotometer, respectively [22]. Furthermore, the ECH2O 5TE Sensor (Decagon devices, Pullman, WA, USA) was deployed for temperature and moisture analyses. Ambient temperature, humidity, and radiation were determined by ATMOS14 (Decagon devices, USA).

### 2.3. Plant Sampling, Fruit Yield, and Yield Attributing Features of Chilli Peppers

Investigations of plant growth parameters were conducted at 30, 50, 70, 100, and 130 days after transplanting (DAT). Harvesting chilli fruits began two weeks after the beginning of the mature stage, around 95 DAT.

The parameters of plant height, dry weight of shoot and root, length of internode, chlorophyll contents of leaf, stem diameter, and number of fruit branches were measured in three replicates. The chilli pepper fruit was harvested every two weeks from each treatment. The number of fruits, fruit weight, and color were recorded until the final harvest. Fruit samples were stored in a −4 °C refrigerator until used for further chemical analyses. Fruit quality attributing parameters, including crude protein and total N, were determined by the modified Kjeldahl method [23]. The yield was calculated by estimating the number of fruits harvested for 130 days.

### 2.4. Statistical Analysis

The effects of organic manure application on growth attributes, nutrient content, and chilli pepper yield were determined under three different temperature conditions in two growing seasons (2017 and 2018), and were subjected to statistical significance using the ANOVA CROPSTAT program. No significant differences in any traits were observed between the two experimental years, and data was pooled before statistical analysis. Student’s t-test was used for comparative analyses between treatments. The mean comparisons were conducted using a least significant difference (LSD) test (*p* ≤ 0.05).

## 3. Results

### 3.1. Effects of Organic Manure and Temperature Increase on the Growth of Chilli Peppers

The application of organic manure had a significant positive effect on plant growth parameters such as plant height, length of internode, number of fruit branches, and stem diameter, and the effect was more pronounced under elevated temperature conditions (Table 2 and Table 3). Organic manure treated at the 2× rate yielded the highest plant growth (plant height, length of internode, number of fruit branches, and stem diameter), was and was substantially higher than those of the 1× rate treatment at all temperature environments. Although a significant difference was not detected, the chlorophyll content of leaf and stem diameter parameters increased with application rate increase. However, compared to the field condition plant growth parameters such as plant height, stem diameter, dry weight of shoot and root, and number of fruit branches tended to be significantly higher under the greenhouse followed by rain-shelter house conditions. The application of organic manure at the 1× rate increased plant height and the number of fruit branch parameters by 28.9% and 37.8% in the greenhouse and by 23.3% and 26.1% in the rain-shelter house, respectively, compared to the field. Likewise, when organic manure was applied at the 1× rate, the length of internode increased by 44.7% and 34.3% in the greenhouse and rain-shelter house, respectively, compared to the field values (Table 2).

**Figure 1:**
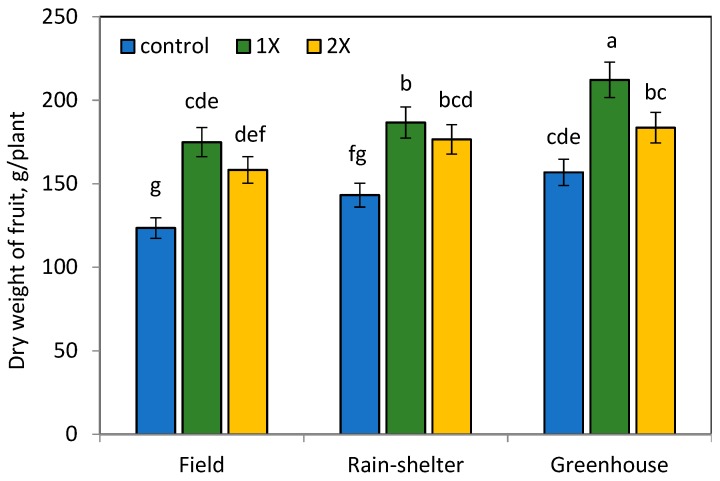
Dry weight of fruit per plant (g) at 130 DAT. Means separated by same lower case letter in each column are not significantly different at *p* < 0.05 among treatments.

**Figure 2:**
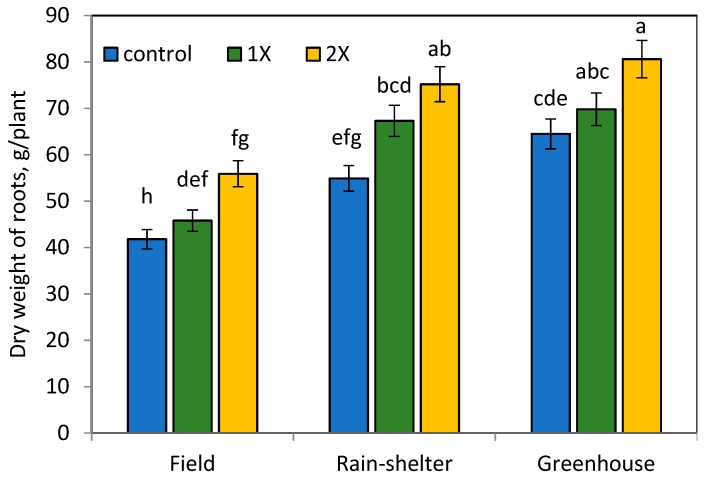
Dry weight of root weight per plant (g) at 130 DAT. Means separated by same lower case letter in each column are not significantly different at *p* < 0.05 among treatments. LSD = least significant difference.

**Figure 3:**
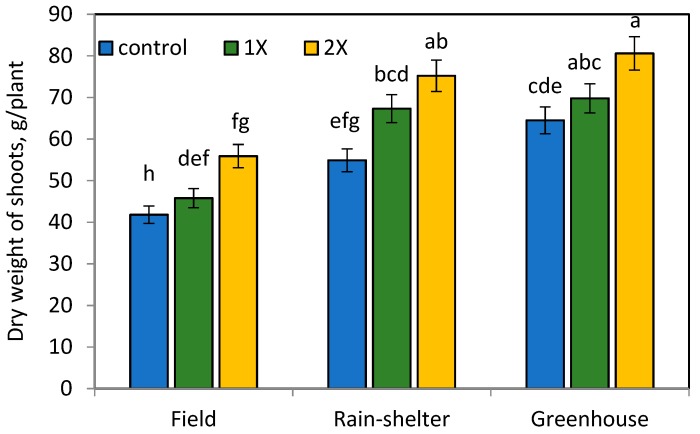
Dry weight of shoot weight per plant (g) at 130 DAT. Means separated by same lower case letter in each column are not significantly different at *p* < 0.05 among treatments.

Similarly, the observation conducted in 130 DAT presented the effectiveness of organic manure on plant growth parameters of chilli pepper (Figure 1, Figure 2 and Figure 3). However, the application of organic manure at the 2× rate significantly increased plant growth attributes, with a slight decrease observed at the 1× rate in all environment conditions.

The enhanced plant development in response to organic manure application is well-known [24], exhibiting a significant steady increase in plant growth due to the improved nutrient conditions in the root rhizosphere. Although organic manure application did not bring any significant difference at the beginning of growing season, the growth parameters were significantly higher at both rates of organic manure treatment at 130 DAT (Table 4). The application of organic manure at the 1× and 2× rates significantly increased chilli pepper growth and N uptake, but the 1× rate treatment stabilized the biomass ratio x plant yield interrelations. However, the organic manure based nutrient management scheme efficiently increased plant growth in all combinations of elevated temperature.

### 3.2. Effects of Organic Manure and Elevated Temperature on Dry Weight and Yield of Chilli Pepper

The highest average of dry weight chilli pepper fruit yield was found at the 1× rate of organic manure application, especially under the greenhouse condition (Figure 1, Figure 2 and Figure 3). Our results show that the dried fruit of chilli pepper at the 1× rate organic manure application was significantly higher by 78.9% in the greenhouse and 20.5% in the rain-shelter house compared to the field condition. Chilli peppers grown in the rain-shelter house at the 2× rate of organic manure treatment showed the highest increase by 24.5%, 34.5%, and 13.5% of root, shoot, and fruit weight attributes, respectively, when compared to the appropriate control in the field. Likewise, root, shoot, and leaf dry weights at the 2× organic manure application in the greenhouse increased by 43.5%, 44.2%, and 36.0%, respectively compared to those of the control values.

However, the highest chilli peppers’ yield was observed at the 1× rate of organic manure application under the greenhouse and followed by rain-shelter house conditions (Figure 3), the nutrient content in the soil probably was enough to meet the requirement of stabilizing crop growth. N sources positively influence crop productivity. However, a higher N input is accompanied by adverse effects for crop yield, soil health, and greenhouse gas emissions [25]. The quantity and quality of organic manure have tremendous effect on N balance of the soil, while other abiotic and biotic factors need to be considered to maintain N efficiency [26]. These results suggest that organic manure application at the 1× rate was sufficient to achieve the maximum chilli pepper yield in the greenhouse followed by the rain-shelter house, compared in the field (Figure 3). The increased temperature in the greenhouse affected the growth of chilli peppers and resulted in the highest yield at the 1× rate of organic manure application, suggesting that there is no need for extra organic manure application. The 2× rate organic manure application compared to the 1× rate caused a 31.3% and 5.8% reduction of fruit yield per plant in the greenhouse and rain-shelter house conditions, respectively. Significantly higher chilli pepper fruit yield harvested in the greenhouse gave a cue that the increased temperature synergistically optimized growth conditions and nutrient availability to produce a maximum chilli pepper yield at the 1× rate of organic manure treatment. The oversupply of organic manure to the soil led to an adverse effect for chilli pepper productivity and decreased its efficiency.

According to recent findings by Gu et al. [27], manure amendment can efficiently reduce ammonia volatilization in different cropping systems. On the other hand, a steady and significant increase in crop yield over the years with the application of organic manures was observed in many previous studies [10]. This might be explained by the fact that the organic manure application at the 2× rate resulted in higher N content in crop vegetative and generative organs than the 1× rate (Table 5, Table 6, Table 7 and Table 8). Furthermore, manure application at the 1× rate sustained a steady and smooth supply of N that might create favorable condition for plant growth and lead to higher N uptake from the soil. In addition, it is reasonable to predict that manure amendments reduce N loss, which might enhance N uptake.

### 3.3. Effects of Organic Manure and Temperature on the Nutrition of Chilli Peppers

The application of organic manure increased plant Ca, K, Mg, Na, total P, and N contents at all temperature environments, especially in the greenhouse condition with significant differences (Table 5, Table 6, Table 7 and Table 8). In the roots, the highest nutrient uptake was observed at the 2× rate of organic manure application in all three temperature conditions. However, the nutrient content in the plant roots was more pronounced in the greenhouse condition (Table 5). Ca, K, and Mg contents increased by 49.8%, 57.8%, and 68.6%, respectively, in the greenhouse condition compared to the respective control in the field condition, while Na and P concentrations were slightly higher in the chilli roots grown under the rain-shelter house condition.

The organic manure application increased nutrient uptake into the roots, thereby nutrient content of the shoots increased (Table 6). Ca, K, and Mg contents of chilli pepper grown at the 2× rate organic manure treatment in the greenhouse condition were higher by 10%, 58.9%, and 17.6% compared to the respective control in the field condition. Similarly, Na, total P, and N contents at the 2× rate of organic manure application were higher by 23.8%, 11.8%, and 7.8%, respectively, compared to their respective control.

A similar trend of increasing nutrient contents by the 2× rate application of organic manure was also detected in the fruit content (Table 7). The increased temperature effect comparing the two rates of organic manure application was significant, suggesting that higher temperature and organic manure application generate greater nutrient content in chilli pepper fruits. Averaged across, organic manure application as a subplot significantly increased total Ca, K, and Mg contents by 11.2%, 12.1%, and 8.7%, respectively, compared to the respective control. A similar trend was observed on total P and N contents in chilli pepper fruits. However, Na content was slightly higher at the 1× rate organic manure application in the greenhouse condition.

Analyses of leaf chemical content of chilli pepper exhibited a similar trend to the increase of nutrient levels depending on the organic manure application (Table 8). The applied organic manure and elevated air temperature interactions promoted nutrient uptake by chilli peppers, which resulted in increased plant biomass and yield parameters. As expected, organic manure application initially improved soil properties and promoted plant growth, which resulted in the increase of plant nutrient uptake, growth, and yield [27]. The application of organic manure and elevated temperature interaction resulted in the abundance of nutrients in the soil, probably associated with the impact of beneficial microbes and favorable conditions for plant growth. These results indicate that organic manure application can alter mineral nutrient profiles in all plant parts, and improve their uptake and mobility within plants.

### 3.4. Effects of Different Rates of Organic Manure Application on the Soil’s Chemical Contents

The application of organic manure significantly increased soil organic matter (OM) and nutrients content in all experimental plots (Table 9 and Table 10). Averaged across soil depths, organic manure application, especially at the 2× rate, significantly increased soil OM content. At the end of the growing season, the highest OM content was observed in the field condition (38.5 mg kg^−1^) followed by greenhouse (37.6 mg kg^−1^), and rain-shelter house (31.5 mg kg^−1^) conditions at the 2× rate of organic manure treatments. Soil pH levels tended to decrease in the field and rain shelter house conditions at the end of the growing season, but slightly increased in the greenhouse condition in all organic manure treatments. The soil’s EC parameter increased in the field condition, but the decrease was observed under the rain-shelter house and greenhouse conditions. Although no significant difference was observed in soil K and Na contents among treatments, some increase was noticed depending on the organic manure application rate, especially in the greenhouse condition. Furthermore, Ca and Mg contents of the soil increased with the increase in organic manure application rate, but it was statistically not significant in most cases.

In the control, OM and N contents slightly decreased at the end of growing season in all temperature conditions. In the case of no organic manure input to the soil, plants uptake available nutrients from the soil and decrease the content of these essential elements. The highest N content in the soil was determined with the highest application rate of organic manure. However, the increasing rates of N in the soil did not exert any significant effects on the concentrations of K and Na, whereas consistently increased Ca and Mg contents. The increase of pepper plant nutrients content and yield was in response to the highly availability of organic nutrients in the soil after organic manure application. Temperature is also known to have a positive effect on the mineralization of soil OM and residues that may have affected nutrient availability in the elevated temperature under greenhouse condition.

It is well documented that higher N fertilization is usually accompanied with increased demand for other macro and micronutrients uptake by plants to maintain a balance. This phenomenon accelerates plant growth and yield formation processes in crops.

## 4. Discussion

Theoretically, incorporating organic manure in soil should improve the growth attributes of plants. However, in this study we demonstrate that an optimum amount of organic manure is advantageous for higher yields. Furthermore, elevated temperatures substantially improved chilli pepper fruit yield in the greenhouse and rain-shelter house conditions. Likewise, plant growth parameters such as the dry weight of leaf, shoot, and root tended to be higher in the greenhouse followed by rain-shelter plastic house compared to the field conditions. Favorable elevated temperature conditions were the reason for the intensive plant growth in the greenhouse, although several authors have declared the negative impact of elevated temperatures on crop productivity [6,28]. However, it is essential to consider the crop’s biological properties and soil-climatic conditions. Chilli peppers originated in Mexico, therefore, this plant prefers high temperatures by its biological properties, and the elevated temperature in the greenhouse may have promoted metabolic processes in the plant cells. Therefore, higher accumulated temperatures of 3091 °C and 3168 °C in the rain-shelter plastic house and plastic greenhouse, respectively, were more effective compared to the lower temperature (2944 °C) in the field (Table 1). Similarly, air and soil temperatures were higher under greenhouse followed by rain-shelter plastic conditions than those in the field condition (Table 1). The accumulated heat from sunlight maintained daily 2–3 °C higher temperature in the greenhouse and rain shelter house during the entire period of the experiment. The elevated air temperature in the greenhouse triggers an increase in soil temperature. This implies that mineralization processes of the applied organic manures in the greenhouse may have been faster than at other ambient conditions.

In the field, the fruit yield did not differ substantially at the 1× and 2× rates of organic manure treatments with values of 179.4 and 158.3 g plant^−1^ at 130 DAT, respectively, with a significant difference compared to the control treatment (123.5 g plant^−1^). On the other hand, these values in the greenhouse condition were substantially higher, recorded 212.2 and 183.6 g plant^−1^ at the 1× and 2× rates of organic manure treatments, respectively. These results show that organic manure along with elevated temperature integration were the main factors that might have stimulated plant growth and chilli pepper yield. The present study is in agreement with results by Guo et al. who reported that elevated temperatures have a positive effect on crop yield in the North China Plain [27].

The organically amended soil is generally reflected in the enhanced chemical content of the plant vegetative parts. The results indicate that the application of organic manure significantly enhances Ca, K, Mg, Na, total P, and N contents in all plant vegetative parts, which is reflected on the growth and yield attributes of chilli peppers. As a subplot effect in this study, an increment in plant growth features was in response to the improved soil quality by the input of organic manure as a fertility amendment. Recently, Surendran et al. reported that increased temperature and moisture cause rapid mineralization of soil organic carbon in tropical regions [29,30]. The results of this study also show that the highest soil moisture was observed in the field followed by greenhouse and rain shelter conditions.

Organic manure application improves soil physical–chemical properties by actively facilitating bacterial growth as well [31,32]. As reported by Gomiero et al., besides enhancing soil quality, organic farming can also improve water use efficiency and this can lead to an increase in yield by 70–90% [2]. In order to have a clear view of organic manure application, a long-term experiment must be conducted. For instance, the experiment conducted in Northeast of China showed that 18 years of manure improved maize yield by 218% when compared to the control value [33]. Furthermore, Diacono and Montemurro found that long-term application of organic amendments increased crop yield by up to 250% and improved crop yield quality [24]. Rational implementation of organic manure is accompanied by the highest productivity of agricultural crops and without environmental damage even under adverse climatic conditions [34,35].

## 5. Conclusions

The beneficial effect of organic manure associated with elevated temperatures was found to have high efficiency in improving chilli peppers’ nutrient content and crop productivity. The application of 2× rate (265.4 kg ha^−1^) organic manure substantially increased the growth parameters of chilli peppers at all temperature conditions, whereas the 1× rate (132.7 kg ha^−1^) exhibited a higher yield increase, especially under elevated temperatures in the greenhouse conditions. Therefore, applying the 1× rate of organic manure is considered the optimum for obtaining a maximum chilli pepper fruit yield. The excessive use of (2× rate) organic manure may result in the accumulation of more vegetative biomass than the increase in chilli pepper yield value. Thus, the rational use of organic amendments for chilli pepper cultivation is highly recommended depending on soil quality and environmental conditions.

Further studies are required to understand the long-term effect of organic manure application on chilli pepper production under variable temperature conditions. Furthermore, it is important to develop a proper simulation model to elucidate the long-term effect of an elevated temperature environment and organic amendments on growth and yield of chilli peppers. Despite forecast uncertainties, the project model allows improving the efficiency of agricultural management practices in various surroundings.

## Figures and Tables

**Table 1:** Weather data on air temperature, rainfall, and relative humidity of the study area, Chungnam province (2017 to 2018 growing seasons’ data).

Year	Month of the Year					
	May	Jun.	Jul.	Aug.	Sep.	Oct.
	Air Temperature (°C)					
2017 Field	22.2	26.4	24.6	24.9	19.5	13.3
Rain-shelter house	22.4	23.7	27.8	26.7	21.8	14.1
Greenhouse	23.3	24.6	28.3	27.1	22.4	14.8
2018 Field	18.8	20.8	25.1	25.1	18.5	12
Rain-shelter house	19.3	21.6	26.6	26.4	19.5	13.1
Greenhouse	20.1	22.4	27.3	26.7	19.7	13.9
	Rainfall (mm)					
2017	35.8	51.4	326.7	358.4	97.1	51.9
2018	90.4	225.5	100	437.1	91.3	90.2
	Relative Humidity (%)					
2017 Field	93.4	94.5	94.9	95.1	95.6	95.7
Rain-shelter house	92.9	93.6	93.8	93.9	94	94.2
Greenhouse	94	94.1	94.6	94.7	95.1	95.2
2018 Field	95.8	95.7	95.8	95.7	96.1	96.5
Rain-shelter house	94.7	94.5	94.6	94.5	94.9	95.3
Greenhouse	95.1	94.9	95.1	95	95.4	95.8
	Soil Moisture					
2017 Field	0.28	0.242	0.221	0.234	0.231	0.224
Rain-shelter house	0.231	0.179	0.166	0.181	0.174	0.166
Greenhouse	0.187	0.193	0.185	0.171	0.185	0.201
2018 Field	0.26	0.264	0.28	0.278	0.279	0.285
Rain-shelter house	0.201	0.174	0.18	0.201	0.196	0.193
Greenhouse	0.249	0.229	0.218	0.233	0.221	0.217

Source: Meteorological Station of Chungnam province, Korea.

**Table 2:** Plant growth attributes at 70 DAT.

Main Plot	Treatments	Height (cm)	Chlorophyll	Length of Internode (cm)	Stem Diameter (mm)	Fruit Branch (No.)
Field	control	86.7 g	62.6 a	6.0 d	9.0 a	22.3 cd
	X1	89.0 g	65.1 a	6.7 cd	9.6 a	23.0 cd
	X2	92.0 fg	68.7 a	8.0 cd	11.1 a	21.0 d
Rain-shelter house	control	106.0 de	66.1 a	8.3 cd	9.1 a	26.3 cd
	X1	109.7 cd	64.6 a	9.0 bc	9.9 a	29.0 bc
	X2	116.7 ab	67.5 a	9.8 ab	10.3 a	24.7 cd
Green house	control	111.0 bc	62.3 a	7.7 cd	10.2 a	31.7 ab
	X1	114.7 bc	62.3 a	9.7 ab	11.0 a	31.7 ab
	X2	122.7 a	65.0 a	10.7 a	11.6 a	38.0 a
LSD 0.05		14.4	6.99	2.16	3.93	7.72
CV (%)		8.2	6.2	8.1	11.6	6.4

Means separated by same lower case letter in each column are not significantly different at *p* < 0.05 among treatments. LSD = least significant difference.

**Table 3:** Dry weights of root, shoot, leaf, and fruits at 70 DAT.

Main Plot	Treatments	Root (g)	Shoot (g)	Leaf (g)	Fruit (g)
Field	control	3.7 g	21.6 g	21.6 e	67.4 g
	X1	4.5 cd	27.6 ef	25.2 e	83.8 de
	X2	4.3 de	23.9 g	22.2 e	77.9 ef
Rain-shelter house	control	4.0 f	31.0 ef	26.4 de	72.3 fg
	X1	4.6 bc	37.8 bc	33.0 bc	95.7 b
	X2	4.7 ab	37.5 cd	34.7 ab	87.2 cd
Green house	control	4.2 ef	34.6 de	23.8 de	85.7 de
	X1	4.7 ab	37.9 ab	31.0 cd	114.6 a
	X2	4.9 a	41.8 a	35.1 a	92.6 bc
	LSD 0.05	2.83	6.45	5.46	10.47
	CV (%)	11.0	13.5	13.1	8.3

Means separated by same lower case letter in each column are not significantly different at *p* < 0.05 among treatments. LSD = least significant difference.

**Table 4:** Plant growth attributes at 130 DAT.

Main Plot	Treatments	Height (cm)	Chlorophyll	Length of Internode (cm)	Stem Diameter (mm)	Branch (No.)
Field	control	88.3 f	67.8 a	7.3 b	13.3 f	26.7 d
	X1	95.0 f	73.1 a	8.7 ab	14.9 ef	28.0 d
	X2	98.7 f	77.3 a	9.0 ab	15.7 de	33.7 cd
Rain-shelter house	control	113.0 de	69.6 a	9.7 ab	14.9 ef	29.3 cd
	X1	116.3 cd	71.7 a	10.0 ab	16.7 bc	35.7 bc
	X2	117.7 bc	71.2 a	10.3 ab	16.1 cd	35.7 bc
Green house	control	110.7 de	69.3 a	9.7 ab	14.7 ef	31.0
	X1	121.3 ab	73.9 a	10.3 ab	17.2 ab	39.0 ab
	X2	126.0 a	74.6 a	11.7 a	17.6 a	42.7 a
LSD 0.05		11.6	6.47	2.92	1.85	8.62
CV (%)		6.0	5.2	17.7	6.9	14.9

Means separated by same lower case letter in each column are not significantly different at *p* < 0.05 among treatments. LSD = least significant difference.

**Table 5:** Root nutrient uptake at 130 DAT.

Main Plot	Treatments	Ca	K	Mg	Na	P	N
		(mg g^−1^)					
Field	control	11.3 h	80.3 c	9.4 cd	3.6 b	3.8 d	46.6 g
	X1	12.9 g	114.9 ab	10.4 cd	2.6 f	3.8 d	51.4 f
	X2	16.2 c	119.8 ab	9.4 cd	2.7 e	4.2 b	52.8 d
Rain-shelter house	control	15.2 e	67.2 c	22.9 a	3.7 b	2.9	36.9 i
	X1	15.5 d	106.8 b	15.7 b	4.1 a	3.8 d	44.5 h
	X2	17.3 a	118.2 ab	11.4 c	3.7 b	4.3 a	56.2 c
Green house	control	14.4 f	75.9 c	20.9 ab	3.1 d	3.3 e	34.2 j
	X1	15.4 d	96.6 bc	15.8 b	3.3 c	4.1c	61.2 b
	X2	16.9 b	126.7 a	15.8 b	2.6 f	4.1c	79.6 a
LSD 0.05		2.3	10.4	4.9	1.3	0.5	0.6
CV (%)		10.4	13.7	13.2	8.1	8.5	8.1

Means separated by same lower case letter in each column are not significantly different at *p* < 0.05 among treatments.

**Table 6:** Shoot nutrient uptake at 130 DAT.

Main Plot	Treatments	Ca	K	Mg	Na	P	N
		(mg g^−1^)					
Field	control	11.1 d	129.9 e	6.1 cd	0.60 bc	3.68 d	44.5 d
	X1	14.4 c	134.6 cd	7.4 c	0.57 bc	3.78 cd	49.9 cd
	X2	25.2 ab	137.9 cd	8.7 ab	0.66 b	4.01 bc	50.1 c
Rain-shelter house	control	18.5 bc	166.3 bc	9.5 ab	0.65 b	3.93 c	44.0 e
	X1	25.8 ab	176.3 b	6.7 bc	0.68 ab	4.12 b	49.7 cd
	X2	27.3 a	166.3 bc	6.9 bc	0.78 a	4.04 bc	50.2 b
Green house	control	20.8 b	155.3 c	7.7 b	0.68 ab	4.04 bc	40.3 f
	X1	24.3 ab	174.1 b	8.1 ab	0.70 ab	4.42 ab	51.7 bc
	X2	27.7 a	219.2 a	10.2 a	0.81 a	4.49 a	54.0 a
LSD 0.05		3.8	29.8	2.48	0.16	0.29	0.57
CV (%)		11.9	12.6	9.6	6.3	4.9	8.1

Means separated by same lower case letter in each column are not significantly different at *p* < 0.05 among treatments.

**Table 7:** Fruit nutrient uptake at 130 DAT.

Main Plot	Treatments	Ca	K	Mg	Na	P	N
		(mg g^−1^)					
Field	control	2.0 f	26.5 bc	1.7 e	1.5 b	3.8 g	22.0 f
	X1	2.7 d	31.8 bc	2.3 c	1.8 a	4.2 d	27.0 c
	X2	2.9 c	34.6 ab	2.5 b	1.8 a	4.3 c	27.9 b
Rain-shelter house	control	2.0 f	26.9 bc	1.7 e	1.2 d	3.9 f	23.5 e
	X1	2.7 d	33.5 ab	2.7 a	1.5 b	3.7 h	27.9 b
	X2	3.2 a	36.9 ab	2.3 c	1.5 b	4.4 b	26.0 d
Green house	control	2.1 e	27.1 bc	1.6 f	0.9 e	4.0 e	21.2 g
	X1	3.1 b	32.2 b	2.1 d	1.4 c	4.2 d	27.1 c
	X2	3.2 a	38.8 a	2.7 a	1.4 c	4.5 a	30.4 a
LSD 0.05		0.43	6.1	0.4	0.4	0.8	0.5
CV (%)		11.2	13.1	12.9	10.7	13.1	13.3

Means separated by same lower case letter in each column are not significantly different at *p* < 0.05 among treatments.

**Table 8:** Leaf nutrient uptake at 130 DAT.

Main Plot	Treatments	Ca	K	Mg	Na	P	N
		(mg g^−1^)					
Field	control	28.9 d	120.1 d	12.2 d	0.66 b	5.1 d	58.2 d
	X1	49.3 bc	182.5 b	15.3 bc	0.59 c	6.7 b	78.6 c
	X2	50.8 b	221.4 a	15.9 b	0.57 c	7.9 a	84.0 a
Rain-shelter house	control	39.3 c	166.2 bc	14.7 c	0.65 b	5.2 d	55.5 e
	X1	59.8 ab	240.5 a	17.2 a	0.77 a	6.7 b	81.6 b
	X2	67.5 a	243.2 a	17.1 a	0.75 a	7.5 ab	79.0 c
Green house	control	49.5 bc	142.6 c	15.0 c	0.73 a	5.3 d	53.2 f
	X1	66.2 a	183.6 b	17.6 a	0.77 a	6.1 c	78.4 c
	X2	67.5 a	243.2 a	17.4 a	0.75 a	7.5 ab	79.0 c
LSD 0.05		11.8	41.1	1.1	0.12	0.5	0.66
CV (%)		15.4	15.0	4.9	15.3	5.2	6.2

Means separated by same lower case letter in each column are not significantly different at *p* < 0.05 among treatments.

**Table 9:** Effects of different rates of organic manure application on soil chemical contents at the end of the experiment.

Main Plot	Treatments	pH (1 : 5)	E.C (dS/m)	OM (g/kg)	NO_3_-N (ppm)	NH_4_^+^-N (ppm)
Field	control	5.8 c	1.2 b	32.4 b	10.7 f	3.1 de
	X1	6.1 b	1.1 b	37.7 a	21.0 d	7.7 c
	X2	6.5 ab	0.7 c	38.5 a	21.5 d	1.6 e
Rain-shelter house	control	6.3 b	1.4 ab	28.6 c	69.0 a	22.7 b
	X1	6.4 ab	1.5 a	31.9 b	51.1 b	23.6 b
	X2	6.8 a	1.4 ab	31.5 b	22.4 d	40.7 a
Green-house	control	5.9 bc	0.8 c	25.6 c	35.8 c	5.7 cd
	X1	6.3 b	1.1 b	31.6 b	19.7 de	4.0 cd
	X2	6.5 ab	1.5 a	37.6 a	17.7 de	7.0 c
LSD 0.05		4.5	0.3	4.6	8.4	4.6
CV (%)		5.1	4.9	5.8	4.8	5.7

Means separated by same lower case letter in each column are not significantly different at *p* < 0.05 among treatments.

**Table 10:** Effects of different rates of organic manure application on soil chemical contents at the end of the experiment.

Main Plot	Treatments	P_2_O_5_, (mg/kg)	K, (cmol/kg)	Ca, (cmol/kg)	Mg, (cmol/kg)	Na, (cmol/kg)
Field	control	374.7 i	1.2 c	7.2 f	1.9 d	0.1 c
	X1	480.6 g	1.2 c	7.8 d	2.5 b	0.1 c
	X2	542.1 f	1.3 bc	7.8 d	2.5 b	0.1 c
Rain-shelter house	control	412.3 h	1.3 bc	8.3 c	2.3 bc	0.2 b
	X1	569.9 e	1.3 bc	8.7 b	2.3 bc	0.2 b
	X2	588.0 d	1.2 c	8.8 a	2.4 bc	0.3 a
Green-house	control	727.6 c	1.3 bc	7.6 e	2.5 b	0.1 c
	X1	772.9 b	1.4 b	8.7 b	2.6 ab	0.2 b
	X2	862.8 a	1.9 a	8.8 a	3.0 a	0.2 b
LSD 0.05		13.4	1.5	0.95	0.5	0.09
CV (%)		6.5	6.3	7.4	9.7	5.6

Means separated by same lower case letter in each column are not significantly different at *p* < 0.05 among treatments.
